# PD-L1 (SP142) testing is concordant between Benchmark Ultra and Bond-III stainers

**DOI:** 10.1007/s00345-021-03828-w

**Published:** 2021-09-13

**Authors:** Eva Compérat, André Oszwald, Gabriel Wasinger, Justine Wacquet, Morgan Rouprêt, Olivier Cussenot

**Affiliations:** 1 GRC n°5, Predictive Onco-Urology, Sorbonne Université, 75020 Paris, France; 2 Department of Pathology, Hôpital Tenon, AP-HP, 4, Rue de la Chine, 75020 Paris, France; 3 Department of Pathology, Medical University of Vienna, Vienna, Austria; 4 Department of Urology, Hôpital Pitié-Salpêtrière, Urology, AP-HP, 75013 Paris, France; 5 Department of Urology, Hôpital Tenon, AP-HP, 75020 Paris, France

**Keywords:** PD-L1 inhibitors, Immunohistochemistry, Bladder cancer, Clinical pathology

## Abstract

**Background:**

Atezolizumab is an inhibitor of programmed death-ligand 1 (PD-L1), used to treat advanced or metastatic bladder cancer, and in trials for non-invasive disease. In order to be eligible for treatment, patients require a PD-L1 immune cell score ≥ 5%, using the Ventana SP142 PD-L1 assay. Many laboratories do not have access to the required Ventana Benchmark Ultra stainer, and it is unclear if the assay performs similarly on other stainers. In this study, we compare SP142 assay results between Ventana Benchmark Ultra and Leica Bond-III stainers.

**Methods:**

Serial sections of 90 samples of transurethral bladder resections (comprising 51 pTaHG, 8 pTis, 18 pT1, 10 pT2 tumors) were stained using the SP142 PD-L1 antibody on Ventana Benchmark Ultra and Leica Bond-III stainers, manually scored, and compared using accuracy and Cohen’s kappa measures.

**Results:**

Both devices yielded highly concordant PD-L1 immune cell scores (accuracy 0.84, Cohen’s κ 0.732). Moreover, we found similar tumor cell (TC) PD-L1 scores using both stainers, and a trend towards greater TC scores in pT2 stage samples (*p* = 0.05).

**Conclusion:**

This study is the first to compare the SP142 antibody in bladder cancer on two different stainers. Our results indicate that both Benchmark Ultra and Bond-III stainers yield highly concordant results using the SP142 PD-L1 antibody.

## Introduction

The European Association of Urology (EAU) and European Society of Medical Oncology (ESMO) recommend Atezolizumab, an inhibitor of programmed death-ligand 1 (PD-L1), as an alternative to carboplatin-based chemotherapy for cisplatin-ineligible patients with treatment-naive locally advanced or metastatic bladder cancer (BC) [1]. Ongoing trials are evaluating the benefit in non-muscle-invasive bladder cancer (NMIBC) as well [2].

The American Food and Drug Administration (FDA) and European Medical Agency (EMA) have approved the use of Atezolizumab for patients with PD-L1 expression in ≥ 5% of immune cells in tumor tissue (IC score) using the Ventana SP142 PD-L1 immunohistochemistry assay [3]. This test is licensed exclusively for performance on the Ventana Benchmark Ultra stainer, which is not available in every laboratory. Since stainers use proprietary reagents, specific protocol timings, and working dilutions, it is unclear if using the SP142 antibody on different stainers yields comparable IC score results.

The aim of this study was to compare the same antibody (SP142, Ventana) on two different commonly available stainers (Ventana Benchmark Ultra and Leica Bond-III) and investigate differences in resulting IC scores. To our knowledge, this is the first study to compare the SP142 antibody in BC on different stainers.

## Material and methods

### Patients and samples

We chose current in-house (Hôpital Tenon, Paris) and referred transurethral resections of bladder (TURB) specimens between the years 2019 and 2020 which were previously stained for PD-L1 in the course of a multicenter BC study where a central review was performed by the corresponding author (EC). Cohort data were collected in Paris at the end of this period and are summarized in the results (Table 1).

**Table 1:** Clinico-pathological characteristics of study cohort. Cis, *carcinoma* in situ

pT stage	Count (%)	Cis (%)	Age (range)	M:F ratio
pTis	8 (8.9)	8 (100)	65 (54–75)	5
pTa	51 (56.7)	12 (23.5)	80 (54–93)	2.3
pT1a	13 (14.4)	8 (61.5)	74 (55–84)	10
pT1b	5 (5.6)	2 (40)	75 (69–85)	3
pT2	10 (11.1)	7 (70)	75.5 (56–94)	4
NA	3 (3.3)	0 (0)	64 (64–77)	1
Total	90 (100)	37 (41.1)	75 (54–94)	3.1

### Histological preparation

Serial sections were cut and stained using HES (hematoxylin, eosin, saffron) for histopathological diagnosis. PD-L1 staining was performed on Benchmark Ultra (Ventana Medical Systems) and Bond-III (Leica Biosystems) stainers using similar protocols outlined as follows. Benchmark Ultra (according to assay instructions): antigen retrieval 4 min at pH 9, incubation with SP142 for 8 min, and counterstaining with hematein for 8 min; Leica Bond-III: antigen retrieval 20 min at pH 9, incubation with SP142 for 15 min, and counterstaining with hematein for 10 min.

Diagnosis of BC and PD-L1 assessment was performed by a senior urogenital pathologist (EC) with training and experience in SP142 PD-L1 assessment standards [4]. Slides from the Benchmark Ultra were scored on a case-by-case basis for diagnostic purposes between 2019 and 2020. Cases were then collectively stained on the Bond-III and scored in a randomized manner, blinded to the results of the Benchmark Ultra. Although not part of the approved SP142 assay, tumor cell (TC) scores were also assessed for the purpose of this study. PD-L1 IC and TC scores were scored in categories of 0–5%, 5–10%, 10–30%, and > 30%.

### Statistical analysis

PD-L1 scores from slides stained on Benchmark Ultra and Bond-III were compared by calculating inter-rater reliability measures, such as accuracy and Cohen’s kappa. Differences between patient groups (such as pT, grade, age, sex) were assessed using analysis of variance. Statistical analysis was performed with RStudio (R Foundation for Statistical Computing, Vienna) using *caret* package [5].

## Results

Ninety cases were included in this study (Table 1). The patients’ median age was 75 years, ranging from 54 to 94 years, with a male-to-female ratio of 3.2:1. The following lesions were assessed in the course of this study: 51 pTaHG (12 with concurrent carcinoma in situ), 8 pTis, 18 pT1 (13 pT1a, 5 pT1b), and 10 pT2. Three cases were reviewed as non-malignant (two cases with hyperplasia of von Brunn epithelial nests and one case of bacillus calmette-guerin-related inflammation). Overall, carcinoma in situ was present in 42% of tumor samples.

**Fig. 1:**
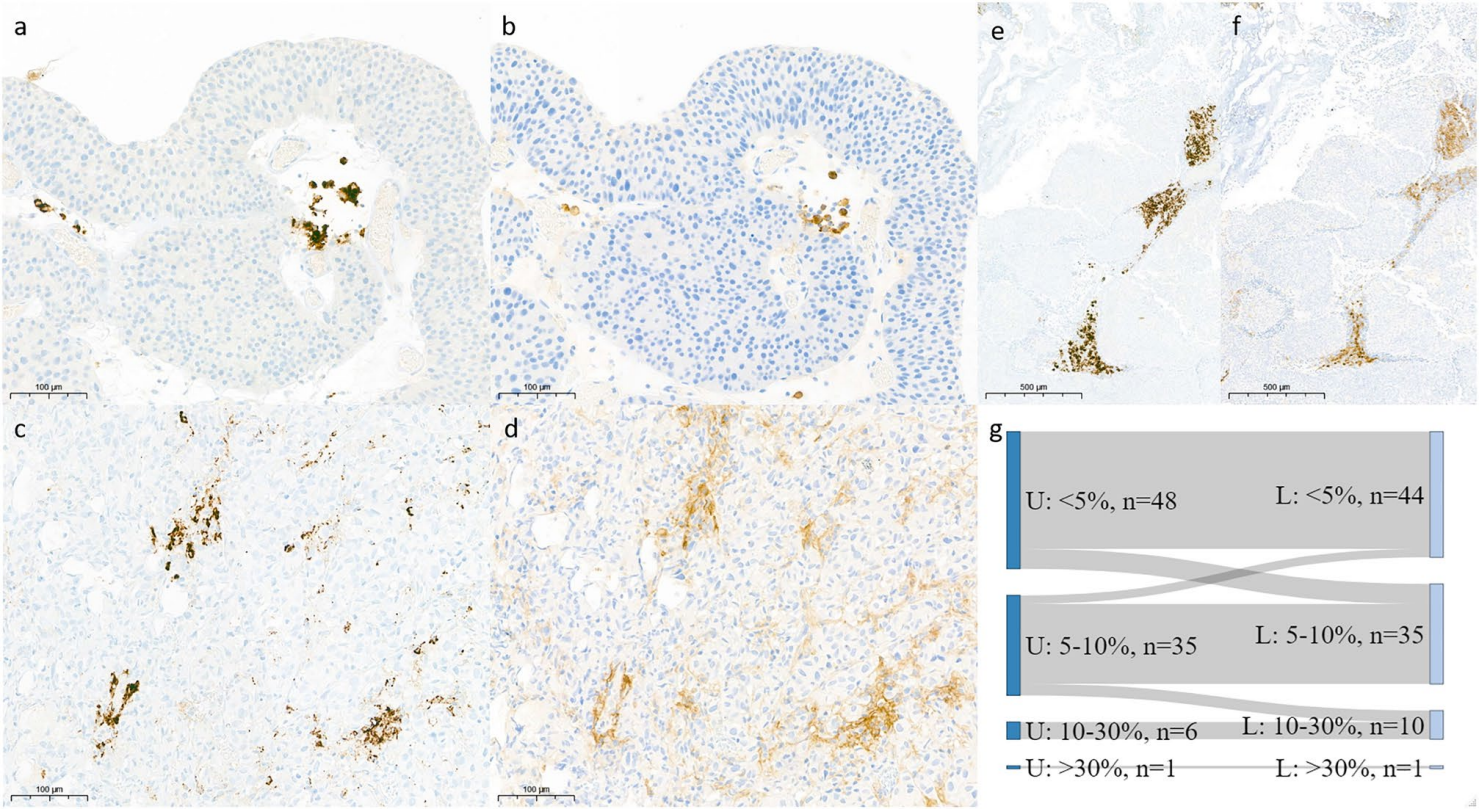
SP142 immunohistochemistry is similar on Ventana and Leica stainers. PD-L1-positive IC in pTa HG tumor stained on Ventana (**a**) and Leica (**b**), 20× DAB. PD-L1-positive IC and TC in an invasive tumor stained on Ventana (**c**) and Leica (**d**), 20× DAB (scale bar, 100 µm). Overview image of PD-L1 staining in pTa tumor stained on Ventana (**e**) and Leica (**f**), 5× DAB (scale bar, 500 µm). Sankey plot describing the difference in PD-L1 scores of samples between sections stained on Ventana (left, U) and Leica (right, L)

Upon comparison of sections stained on Benchmark Ultra and Bond-III, we observed that the resulting staining patterns were very similar but more intense and less granular resulting from the Bond-III (Fig. 1a-f). Samples from both stainers yielded highly concordant IC scores (Table 2). Roughly half of all cases had IC scores < 5% (Benchmark Ultra: 52%, Bond-III: 49%). In total, 14/90 cases (15.6%) were scored in a different group using the Bond-III compared to the Benchmark Ultra. Importantly, only three cases (3.3%) that surpassed the IC ≥ 5% threshold on the Benchmark Ultra scored IC < 5% on the Bond-III (Fig. 1g). Vice versa, seven (7.7%) cases that were scored IC < 5% on the Benchmark Ultra were scored between 5 and 10% on the Bond-III.

**Table 2:** IC and TC scores on Ventana Benchmark Ultra and Leica Bond-III stainers showing high concordance

IC score	Ventana Benchmark Ultra					Sum
		< 5%	5–10%	10–30%	> 30%	
Leica Bond-III	< 5%	41	3	0	0	44
	5–10%	7	28	0	0	35
	10–30%	0	4	6	0	10
	> 30%	0	0	0	1	1
Sum		48	35	6	1	

TC score	Ventana Benchmark Ultra					Sum
		< 5%	5–10%	10–30%	> 30%	
Leica Bond-III	< 5%	71	0	0	0	71
	5–10%	6	7	0	0	13
	10–30%	1	3	1	0	5
	> 30%	0	0	0	1	1
Sum		78	10	1	1	

The accuracy of the assay on the Bond-III compared to the Benchmark Ultra was 0.84 (significantly greater than the no-information rate, *p* = 4.7^-10). Cohen’s kappa comparing overall results from the two stainers was 0.732. Compared to the standard assay performed on the Benchmark Ultra, the sensitivity and specificity of the assay performed on the Bond-III to identify samples with IC ≥ 5% were 0.85 and 0.93, respectively. Upon sub-analysis of the 10 pT2 samples, the assay accuracy on the Bond-III compared to the Benchmark Ultra was 0.9 (significantly greater than the no-information rate, *p* = 0.0017) with Cohen’s kappa = 0.86, slightly higher than for the overall cohort. Thus, we observed highly concordant staining results between the two stainers, with a trend towards overestimation using the Bond-III. SP142 IC scores did not significantly differ between tumor stages, patient age, or gender (data not shown).

Tumor cell (TC) scores are not part of the SP142 assay. However, in order to obtain a more detailed comparison between stainers, TC scores were assessed as well. Again, we observed highly concordant results (Table 1). TC scores between 5 and 10% were obtained for 85% of samples stained with the Benchmark Ultra and 79% of samples stained by the Bond-III. The accuracy of the Bond-III TC score in recapitulating Benchmark Ultra TC score was 0.89; Cohen’s kappa was 0.629.

One extremely unusual case of BC (pT2) displayed PD-L1 staining in > 30% of IC and TC on both stainers. Even when excluding this case from analysis, we found a trend towards higher Benchmark Ultra TC scores in pT2 tumors (ANOVA: *p* = 0.019, Tukey’s HSD: *p* = 0.05), but no differences in patient age or gender (data not shown).

## Discussion

In this study, we compared one PD-L1 antibody clone (Ventana SP142) on Ventana Benchmark Ultra and Leica Bond-III stainers. Using two similar staining protocols, we obtained highly concordant results in terms of visual appearance and IC scores, both in our overall cohort and in sub-analysis of only pT2 cases. We observed a slightly stronger and less granular signal in slides stained on the Bond-III, and conclude that this does not affect interpretation of the samples. Nonetheless, our data suggest a trend towards higher scores when stained with the Bond-III; additional refinement of the protocol (i.e., shorter antigen retrieval) could yield even closer results. Lastly, we found a trend towards higher TC scores in pT2 tumors (*p* = 0.05, Benchmark Ultra).

Recent publications have indicated interchangeability of several diagnostic PD-L1 antibodies. [6, 7]. Others have shown that the inter-rater variability is greater than the variability between different antibody clones [8]. In one study, it was hypothesized that certain staining characteristics of the SP142 assay might be attributed to the specific composition of the test reagents and properties of the Benchmark Ultra [9]. Our study shows that although the staining pattern is less granular and slightly stronger using the Bond-III, the overall staining results are comparable.

Limitations of this work include the small cohort size and the single-observer analysis, underlining the preliminary nature of this study. Further work is necessary to systematically evaluate different antibodies on different platforms in order to make more general statements. Moreover, this study involved mostly cases of NMIBC, for which Atezolizumab is currently not approved; lastly, we do not show the frequency of metastasis in our cohort. However, ongoing clinical trials are testing the efficacy of PD-L1 inhibitors in NMIBC and we expect our current results to become more relevant in the near future.

In conclusion, we compared the staining of a commonly used PD-L1 antibody clone (SP142) on two different immunohistochemistry stainers (Ventana Benchmark Ultra and Leica Bond-III) using BC tissue and report highly concordant results, suggesting that the test could be performed on other stainers besides the licensed Benchmark Ultra. This finding could enable access to diagnostic tests—and consequently, immune checkpoint inhibitor treatment—in laboratories where a Benchmark Ultra stainer is not available.

## Data Availability

All data are in the hands of authors EC and AO.

## References

[1] Horwich A, Babjuk M, Bellmunt J, Bruins HM, De Reijke TM, De Santis M (2019). EAU–ESMO consensus statements on the management of advanced and variant bladder cancer—an international collaborative multi-stakeholder effort: under the auspices of the EAU and ESMO Guidelines Committees†. Ann Oncol.

[2] Roupret M, Neuzillet Y, Bertaut A, Pignot G, Houede N, Champiat S (2019). ALBAN: an open label, randomized, phase III trial, evaluating efficacy of atezolizumab in addition to one year BCG (bacillus Calmette-Guerin) bladder instillation in BCG-naive patients with high-risk nonmuscle invasive bladder cancer (AFU-GETUG 37). J Clin Oncol.

[3] Teo MY, Rosenberg JE (2018). EMA and FDA prune the checkpoint inhibitor treatment landscape. Nat Rev Urol.

[4] Eckstein M, Cimadamore A, Hartmann A, Lopez-Beltran A, Cheng L, Scarpelli M (2019). PD-L1 assessment in urothelial carcinoma: a practical approach. Ann Transl Med.

[5] Kuhn M (2020) caret: Classification and Regression Training [Internet]. https://CRAN.R-project.org/package=caret. Accessed 14 July 2021

[6] Tretiakova M, Fulton R, Kocherginsky M, Long T, Ussakli C, Antic T (2018). Concordance study of PD-L1 expression in primary and metastatic bladder carcinomas: comparison of four commonly used antibodies and RNA expression. Mod Pathol.

[7] Schwamborn K, Ammann JU, Knüchel R, Hartmann A, Baretton G, Lasitschka F (2019). Multicentric analytical comparability study of programmed death-ligand 1 expression on tumor-infiltrating immune cells and tumor cells in urothelial bladder cancer using four clinically developed immunohistochemistry assays. Virchows Arch.

[8] Brunnström H, Johansson A, Westbom-Fremer S, Backman M, Djureinovic D, Patthey A (2017). PD-L1 immunohistochemistry in clinical diagnostics of lung cancer: inter-pathologist variability is higher than assay variability. Mod Pathol.

[9] Schats KA, Van Vré EA, Boeckx C, De Bie M, Schrijvers DM, Neyns B (2018). Optimal evaluation of programmed death ligand-1 on tumor cells versus immune cells requires different detection methods. Arch Pathol Lab Med.

